# Reconstruction of Complex Lateral Skull Base Defects After Oral Cancer Resection With Individualized Anterolateral Thigh Flap

**DOI:** 10.3389/fonc.2021.743370

**Published:** 2021-09-22

**Authors:** Zhaojian Gong, Shanshan Zhang, Chang Chen, Yuan Zhi, Moxin Zi

**Affiliations:** ^1^Department of Stomatology, The Second Xiangya Hospital, Central South University, Changsha, China; ^2^Department of Stomatology, Xiangya Hospital, Central South University, Changsha, China

**Keywords:** oral cancer, lateral skull base, anterolateral thigh flap, defect, reconstruction

## Abstract

**Objectives:**

Complex lateral skull base defects resulting from advanced or recurrent oral cancer resection are continuously challenging reconstructive surgeons. This study aimed to use reconstructive methods for lateral skull base defects, explore their feasibility, and evaluate the efficacy of defect reconstruction using anterolateral thigh (ALT) flaps.

**Patients and Methods:**

We performed a retrospective case series of 37 patients who underwent lateral skull base defect reconstruction using the ALT/anteromedial thigh (AMT) flap between March 2016 and May 2021 at the Second Xiangya Hospital. The design and harvest of the flaps, methods for defect reconstruction, and reconstructive efficacy are described.

**Results:**

Of the 37 patients, 3 were women and 34 were men, with a mean age of 51.7 years. Among the defects, 26 were through-and-through defects and were reconstructed using ALT chimeric flaps, double ALT flaps, folded ALT flap, combined ALT chimeric flaps and AMT flaps, or combined ALT chimeric flaps and pectoralis major flaps; the large lateral skull base dead spaces were filled with muscle tissues or fatty tissues. Postoperatively, 38 of the 39 ALT/AMT flaps survived completely, and the remaining flap experienced partial necrosis. Venous compromise occurred in one patient who was salvaged after operative exploration. Oral and maxillofacial wound infections occurred in two patients, salivary fistula in three patients, and thigh wound effusion in three patients. The wounds healed gradually in all patients after repeated dressing changes. Thirty-three patients were followed up for approximately 3–60 months; their oral functions and appearance were acceptable, and thigh motor dysfunction was not observed.

**Conclusions:**

With the convenient flap design and muscle flap harvest, large and individualized tissue supply, feasible combination with other flaps, effective reduction or avoidance of wound complications, and acceptable donor site morbidity, the ALT flap is an appropriate choice for complex lateral skull base defect reconstruction.

## Introduction

Oral cancer is a group of malignant diseases arising from the surface of the tongue, gums, buccal mucosa, floor of the mouth, palate, and lips (1, 2). Current therapies for oral cancer include surgery, radiotherapy, chemotherapy, and combination therapy (1, 3). In general, surgery is the primary treatment modality in most cases. The wide local excision of tumors often results in large and complex oral and maxillofacial defects, severely damaging the function and appearance and even leading to psychological disorders (4). In particular, complex multitissue defects resulting from the radical resection of some recurrent or locally advanced-stage tumors may also involve the lateral skull base. Varying-sized dead spaces are often left in these lateral skull base defects, leading to wound infection or effusion and even more serious complications (4). The reconstruction of such defects remains a reconstructive challenge because of the limited local tissue supply and extremely visible location (5–7).

Several free or pedicle flaps, such as radial forearm flap (8), latissimus dorsi flap (9), anterolateral thigh (ALT) flap (4, 10–12), and pectoralis major flap (13), have been used to repair oral and maxillofacial soft-tissue defects. Among them, the ALT flap, which was first introduced in 1984 by Song et al. (14), is a good candidate. With noticeable advantages and acceptable donor site morbidity, this flap has been increasingly applied in recent years (5, 7, 15–17). The ALT flap is nourished by the lateral circumflex femoral artery (LCFA), and the classic ALT flap or its chimeric flaps are pedicled with the descending branch (DB), the largest and longest branch of the LCFA (4, 17). Therefore, various-sized muscles or adipose tissue can be raised simultaneously with the ALT flap, which is crucial for dead space filling and beneficial for wound healing (4).

ALT flaps have been used to repair lateral skull base defects for some time in our department; they are suitable for the reconstruction of such large and complex defects, which are often caused by extensive resection of recurrent or advanced oral cancer. With the large muscle and/or adipose tissue for dead space filling, reconstruction can result in acceptable esthetic and functional outcomes as well as reduced postoperative complications. Herein, we report our experience with 37 patients who underwent complex lateral skull base defect reconstruction using ALT/anteromedial thigh (AMT) flaps or their chimeric flaps from March 2016 to May 2021.

## Patients and Methods

### Study Design

We designed and implemented a retrospective study to address our research aims. The study population included all patients who presented for management and evaluation of lateral skull base defects and underwent reconstruction using the ALT flap or its chimeric flaps from March 2016 to May 2021 at the Second Xiangya Hospital of Central South University. The inclusion criteria of the study included the following: defects involving the lateral skull base, defects resulting from the resection of locally advanced-stage or recurrent tumors, and defects repaired using ALT flaps or combined ALT flap and other flaps. Moreover, the exclusion criteria were defects not caused by tumor resection or those not reconstructed using ALT flaps. The hospital institutional review board approved this project, and the study followed the guidelines set forth in the Declaration of Helsinki.

### Surgical Technique

**Figure 1:**
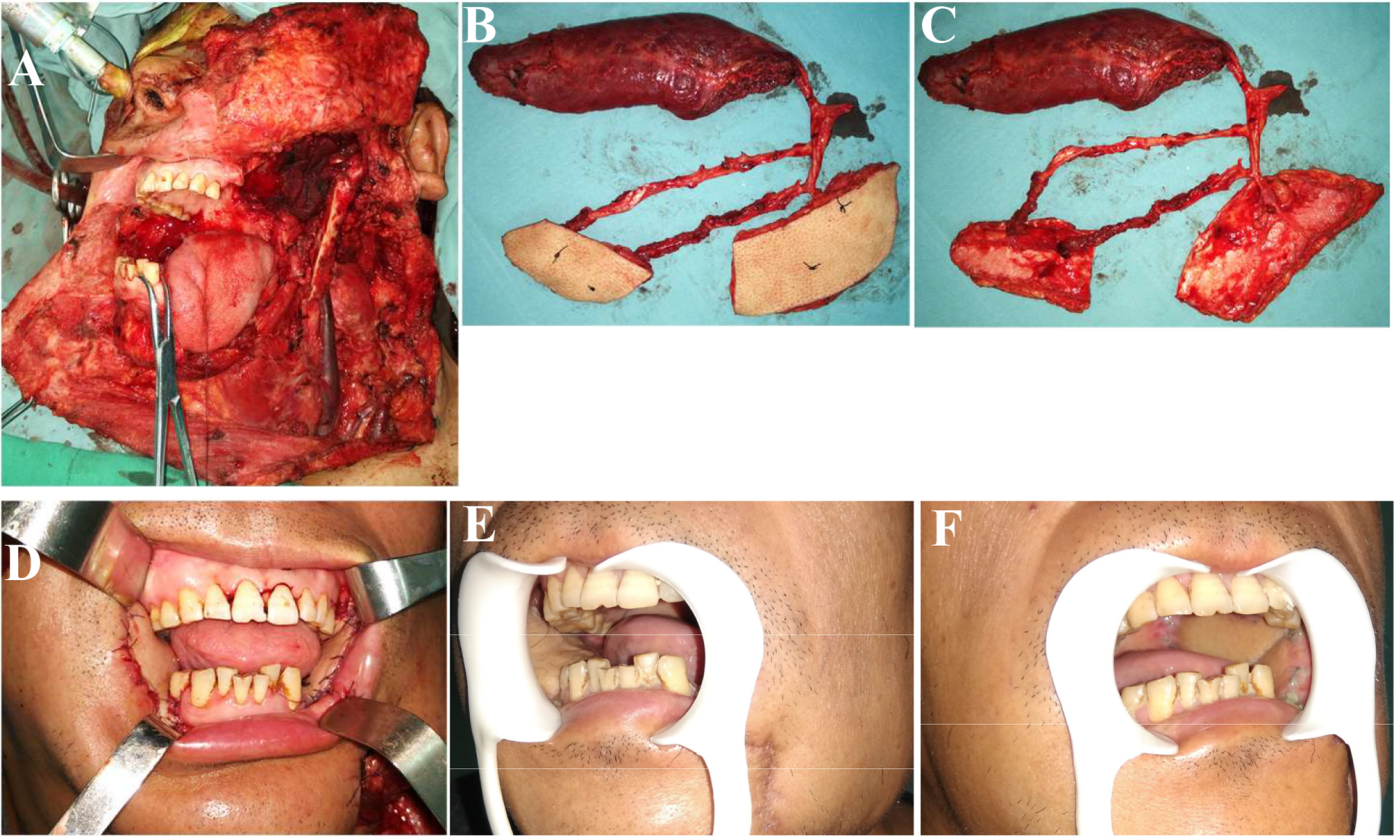
Reconstruction of complex bilateral buccal mucosal defects (involving the lateral skull base) using ALT chimeric flaps. **(A)** Left lateral skull base defects and right buccal mucosal defects resulting from bilateral buccal squamous cell carcinoma resection. **(B, C)** Chimeric ALT, ALT, and rectus femoris flaps (entire rectus femoris). **(D)** Reconstruction of complex defects using ALT chimeric flaps, separate flaps for bilateral buccal mucosal reconstruction, and rectus femoris flap for lateral skull base dead space filling. **(E, F)** One month postoperatively. ALT, anterolateral thigh.

**Figure 2:**
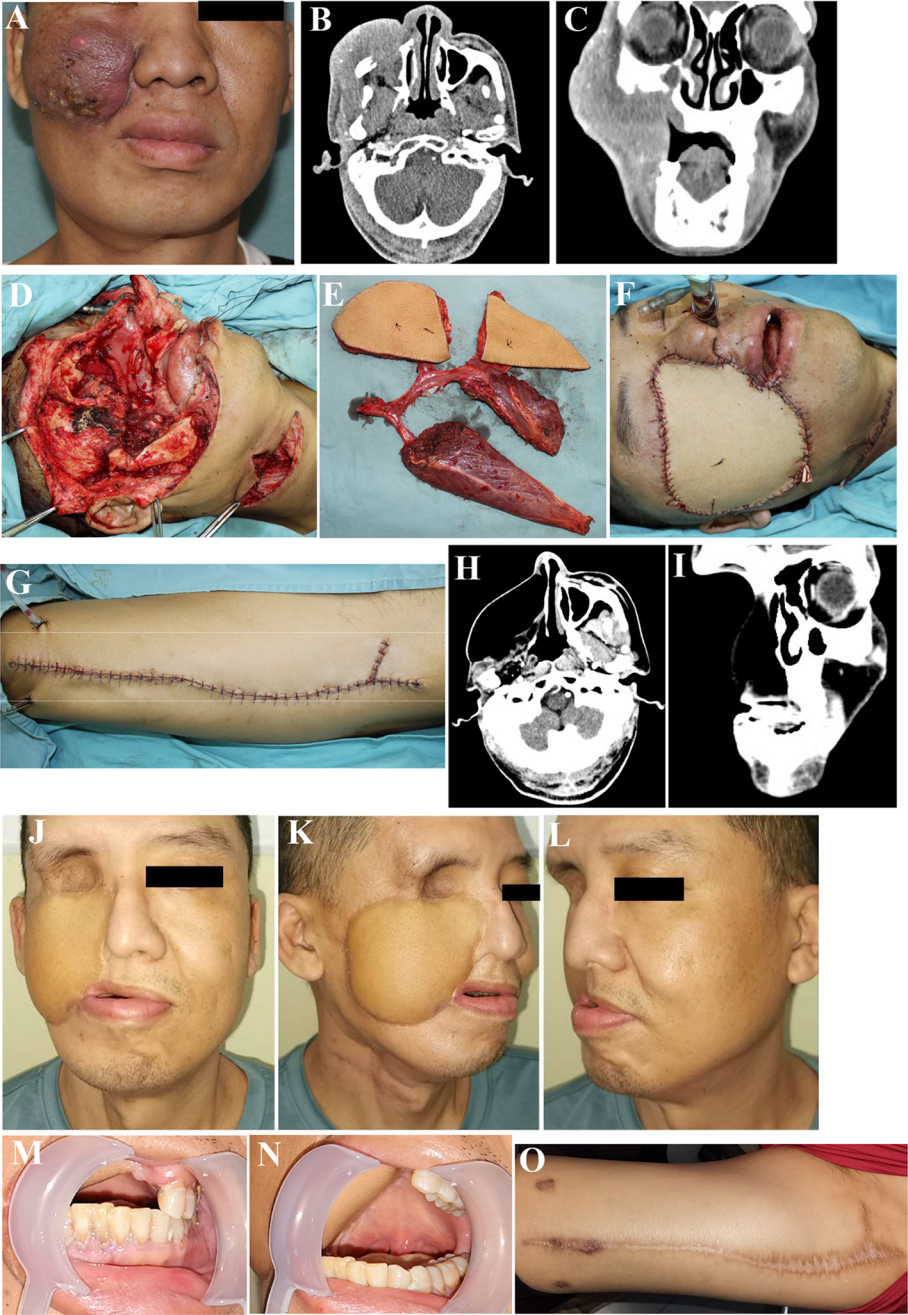
Reconstruction of complex lateral skull base defects using ALT chimeric flaps. **(A)** Recurrence of maxillary gingival squamous cell carcinoma, postoperatively after radiation therapy. **(B, C)** Preoperative CT. **(D)** Through-and-through cheek and lateral skull base defects after tumor resection. **(E)** Chimeric ALT, ALT, and rectus femoris flaps (entire rectus femoris). **(F)** Defects reconstruction using ALT chimeric flaps, separate flaps for intraoral mucosal and extraoral skin reconstruction, and rectus femoris flap for lateral skull base dead space filling. **(G)** Primary closure of the donor site. **(H, I)** Postoperative CT, 52 months postoperatively. **(J–O)**. Sixty months postoperatively. ALT, anterolateral thigh.

The flaps were harvested simultaneously with tumor ablation and/or neck dissection in two groups, and the methods for flap design and elevation were as previously described (4, 17). After sectioning of the skin, subcutaneous fat, and fascia, the lateral and/or medial fascia lata were opened, and sizable cutaneous perforators were explored for the ALT and/or AMT flaps. The ALT flaps or their chimeric flaps were harvested accordingly, with the flaps aimed at reconstructing intraoral mucosal and extraoral skin defects and the muscle and/or fat tissue at filling the lateral skull base dead space (**Figures 1**, **2**). Through-and-through defects were repaired using ALT chimeric flaps, double ALT flaps, folded ALT flap, and combined ALT chimeric flaps and AMT flaps and very large defects using combined ALT chimeric flaps and pectoralis major flaps. For some patients, the entire rectus femoris was harvested to fill the dead spaces, which were located in the temporal, subzygomatic, lateral skull base, and submandibular regions. Additionally, the individualized muscle flaps were harvested with the distal end of the vascular pedicles, which are usually the DB and long enough for all oral and maxillofacial dead spaces. Postoperatively, all flaps were strictly and carefully monitored as previously described, and salvage surgery was immediately performed when flap compromise occurred (12).

## Results

Among the 37 patients, 3 were women and 34 were men, with a mean age of 51.7 years (range, 40–64 years). At presentation, 25 patients had buccal squamous cell carcinoma (SCC), 1 patient had mandibular SCC, 1 patient had palate SCC, 1 patient had gingival SCC, and 9 patients had recurrent oral SCC.

All 37 complex defects involving the lateral skull base, including 26 through-and-through defects, were repaired using 39 ALT/AMT flaps or their chimeric flaps and 1 pectoralis major flap. The skin paddles of the ALT/AMT flaps were 5 × 9−11 × 14 cm in size. The through-and-through defects were repaired using ALT chimeric flaps in 22 cases, double ALT flaps in 1 case, folded ALT flap in 1 case, combined ALT chimeric flaps and AMT flaps in 1 case, and combined ALT chimeric flaps and pectoralis major flaps in 1 case, with separate flaps or folded flap for extraoral skin and intraoral mucosal reconstructions. The muscle and fat tissue, such as the rectus femoris and vastus lateralis flaps, were individually designed and harvested to fill the temporal, lateral skull base, subzygomatic, and submandibular dead spaces. The entire rectus femoris was harvested in 20 patients.

Postoperatively, 38 of the 39 ALT/AMT flaps and 1 pectoralis major flap survived completely, and the remaining ALT flap experienced partial necrosis. Venous compromise occurred in 1 patient, which was salvaged after operative exploration, and partial necrosis of the flap then occurred. Of the donor sites (thigh and chest), 35 were primarily closed, resulting in only linear scars, and the other 2 (thigh) were closed using full-thickness skin grafts due to larger defects. In 1 of these 2 cases, the chimeric ALT plus ALT flaps (nourished by the LCFA) and AMT flap (supplied by a branch of the femoral artery) were elevated in the same thigh, and they were then combined in tandem *via* additional vascular anastomoses and used for large defect reconstruction. Oral and maxillofacial wound infections occurred in 2 patients, salivary fistula in 3 patients, and thigh wound effusion in 3 patients. The wounds healed gradually in all patients after repeated dressing changes.

Thirty-three patients were followed up for approximately 3 to 60 months, and their oral functions and appearance were acceptable. Thigh scars caused by flap harvest were not readily visible, and thigh dyskinesia was not observed. Tumor recurrence and/or metastasis occurred in 16 patients during follow-up. Of them, 12 patients received non-surgical treatment or refused further treatment, and the other 4 patients underwent reoperation, including tumor resection and/or contralateral neck dissection.

## Discussion

The reconstruction of massive defects resulting from locally advanced-stage or recurrent oral cancer resection is still a difficult challenge owing to the limited local tissue supply, extensive skin and soft-tissue defects, and especially the complex oral and maxillofacial structures, including the existence of the zygomatic bone, zygomatic arch, maxilla, mandible, and other bone tissues (4–6). Large dead spaces are often left in defects involving the lateral skull base, easily leading to wound infection or effusion and even other potentially life-threatening complications (4). Owing to the convenient and individualized flap design, large skin territory, sufficient soft-tissue supply, long pedicle and suitable vessel caliber, minimal and acceptable donor site morbidity, and high success rate of transplantation, the ALT flap has been referred to as a “versatile soft-tissue flap” (4, 18). Many groups, including ourselves, have considered the ALT flap or its chimeric flaps as the preferred choice for oral and maxillofacial soft-tissue defect reconstruction after tumor resection (7, 15).

The wide surgical excision of oral cancer often results in large dead spaces and may lead to wound effusion or infection. In particular, for some locally advanced-stage or recurrent tumors, defects often involve the lateral skull base and thus complicate defect reconstruction. These dead spaces, including lateral skull base defects, usually require sufficient muscle and/or fat tissue to be filled. The use of ALT flaps or their chimeric flaps for various defect reconstructions can be accomplished *via* incorporating different soft-tissue components, including the skin, fat, fascia, muscle, and nerve (18, 19). According to our previous reports, muscle tissues of various sizes, including the rectus femoris, vastus medialis, and vastus lateralis, could be easily raised with the flap (4, 17). Moreover, the individually designed fat flaps can also be harvested concomitantly or independently, with the flap or other cutaneous perforators originating from the same vascular pedicle (5, 17). This is excellent for the reconstruction of complex oral and maxillofacial defects in which varying-sized dead spaces are often included. The long vascular pedicle, which permits better convenience and freedom in defect reconstruction, is an obvious advantage of the ALT flap (6, 7). With the long pedicle and individually designed muscle flap and/or fat flap, almost all of the dead spaces resulting from oral cancer ablation, such as the temporal region, lateral skull base, subzygomatic region, submandibular region, and floor of the mouth, can be well filled. In our experience, the rectus femoris branch vessel, which arises from the DB of the LCFA and nourishes the rectus femoris, is relatively constant; thus, the elevation of the rectus femoris flap is safe and straightforward (4, 20). With the separated pedicle, rectus femoris branch vessel, and a large amount of muscle tissue with a sufficient length, the rectus femoris flap could be flexibly used either to fill the dead spaces in any position of the oral and maxillofacial region or to cover the major cervical vessels and/or titanium plate (4). Additionally, the ALT flaps were mainly pedicled with the DB, the longest branch of the LCFA; thus, the muscle flaps that elevated with the distal end of the pedicles can also be used flexibly, similar to the rectus femoris flaps. This is particularly useful for the temporal region, subzygomatic region, and lateral skull base reconstructions in which the temporalis muscle is completely removed because of the tumor. In our series, all dead spaces were obliterated with sufficient muscle and/or fat tissue, and the entire rectus femoris was used in 20 patients. Only 2 and 3 patients developed wound infection and salivary fistula, respectively, which eventually healed following repeated dressing changes. One case of wound infection was caused by flap compromise and partial necrosis.

The reconstruction of lateral skull base defects after oral cancer resection is cosmetically, technically, and functionally challenging, especially when presented with through-and-through defects. In addition to the three-dimensional dead spaces that require sufficient muscle and/or fat tissue, the complex intraoral mucosal and extraoral skin defects also require large and separated skin paddles. Although single skin paddle, such as folded ALT flap, can also repair these defects, good outcomes cannot always be accomplished, particularly if the defects are very large or the corners of the mouth are excised (5). The use of separated skin paddles to repair extraoral skin and intraoral mucosal defects can effectively improve the reconstruction, with acceptable appearance, good functions, and a high degree of patient satisfaction (5). According to our experience, ALT chimeric flaps can be easily raised to reconstruct through-and-through defects (17). Chimeric ALT plus AMT flaps or chimeric ALT plus ALT flaps can be designed according to the location of the cutaneous perforators in the thigh. Although ALT chimeric flaps are a good candidate for through-and-through defect reconstruction, chimeric flaps cannot always be successfully elevated in some patients. Double ALT flaps can also be used. They can either be combined with each other through additional vascular anastomosis or be separately used with the respective vascular anastomosis to the cervical vessels. In cases in which the defects are very large, the ALT flap or its chimeric flaps can also be combined with other flaps, such as the pectoralis major flap. Among the 26 through-and-through defects in our series, 22 were reconstructed using ALT chimeric flaps, 1 using double ALT flaps, 1 using folded ALT flap, 1 using combined ALT chimeric flaps and AMT flaps, and 1 using combined ALT chimeric flaps and pectoralis major flaps. With separated skin paddles for the reconstruction of extraoral skin and intraoral mucosal defects, the functional and esthetic outcomes are acceptable. Moreover, the combined ALT flap and pectoralis major flaps can serve as a good choice for the reconstruction of very large oral and maxillofacial through-and-through defects.

Sometimes, a few patients may present with multiple oral mucosal lesions, resulting in complex multiple and non-adjacent defects. The reconstruction of such complex defects remains a major surgical challenge because of the need for flexible and feasible chimeric flaps (21). Although double or more flaps from different donor sites can meet the requirements, the operating time, difficulty, and local damage are increased due to the need of additional donor sites (5). Moreover, two or more sets of recipient vessels are needed; thus, this strategy cannot be achieved in cases with a single available recipient vessel (6). With the long pedicle and consequent freedom in position, the different skin paddles of ALT chimeric flaps can usually be separated at a distance; thus, these flaps can be used to repair multiple non-adjacent defects. As shown in **Figure 1**, ALT chimeric flaps were successfully used to reconstruct bilateral buccal mucosal defects. The use of ALT chimeric flaps to repair multiple and non-adjacent oral and maxillofacial defects not only resulted in reconstruction with acceptable appearance and oral functions but also effectively avoided the use of additional donor sites, thus reducing local damage.

One of the prominent advantages of the ALT flap over other flaps is reduced and acceptable donor site morbidity (6, 19, 22). Normally, thigh donor site defects narrower than 8 cm and even defects larger than 8 cm can be closed directly (6). In our series, only 2 thigh donor sites were closed using full-thickness skin grafts due to larger defects, while the other 35 donor sites were primarily closed, resulting in only linear scars. In 1 patient, the skin graft was raised from the upper part of the same donor site without additional donor sites. In the other patient, in whom the chimeric ALT plus ALT flaps and AMT flap were elevated in the same thigh, the skin graft was harvested from the abdomen. All donor sites healed well, without significant morbidity. The harvest of the rectus femoris did not significantly increase thigh motor dysfunction, without patient complaints regarding inconvenience in daily life. In addition, it is interesting that such harvest, which reduces the amount of thigh tissue, is conducive to the direct suture of donor site defects (4).

Any type of flap, including the ALT flap, has disadvantages. The harvest of the ALT flap is often more complex and riskier than that of other flaps because of the complicated perforator dissection and high perforator variation. In addition, the ALT chimeric flaps with separated skin paddles cannot always be successfully harvested owing to the lack of adequate cutaneous perforators.

## Conclusions

With the convenient flap design and muscle flap harvest, large and individualized tissue supply, feasible combination with other flaps, effective reduction or avoidance of wound complications, and acceptable donor site morbidity, the ALT flap is an appropriate choice for complex lateral skull base defect reconstruction.

## Data Availability Statement

The original contributions presented in the study are included in the article/supplementary material. Further inquiries can be directed to the corresponding authors.

## Ethics Statement

The studies involving human participants were reviewed and approved by the Ethics Committee of the Second Xiangya Hospital, Central South University. Written informed consent for participation was not required for this study in accordance with the national legislation and the institutional requirements. Written informed consent was obtained from the individual(s) for the publication of any potentially identifiable images or data included in this article.

## Author Contributions

Study concepts and design: ZG and SZ. Data acquisition: CC, YZ, and MZ. Quality control of data and algorithms: CC, YZ, and MZ. Data analysis and interpretation: ZG, CC, YZ, and MZ. Manuscript preparation and editing: ZG, SZ, and CC. All authors contributed to the article and approved the submitted version.

## Conflict of Interest

The authors declare that the research was conducted in the absence of any commercial or financial relationships that could be construed as a potential conflict of interest.

## Publisher’s Note

All claims expressed in this article are solely those of the authors and do not necessarily represent those of their affiliated organizations, or those of the publisher, the editors and the reviewers. Any product that may be evaluated in this article, or claim that may be made by its manufacturer, is not guaranteed or endorsed by the publisher.
